# Cognitive Behavioural Therapy for schizophrenia - outcomes for functioning, distress and quality of life: a meta-analysis

**DOI:** 10.1186/s40359-018-0243-2

**Published:** 2018-07-17

**Authors:** Keith R. Laws, Nicole Darlington, Tejinder K. Kondel, Peter J. McKenna, Sameer Jauhar

**Affiliations:** 1 School of Life and Medical Sciences, University of Hertfordshire, College Lane Campus, Hatfield, AL10 9AB UK; 2 East London Foundation Trust, London, UK; 3 FIDMAG Germanes Hospitalàries Research Foundation, Barcelona and CIBERSAM, Barcelona, Spain; 4 Centre of Affective Disorders, Institute of Psychiatry, London, UK

**Keywords:** Schizophrenia, Psychosis, CBT, CBTp, Cognitive behavioural therapy, Meta-analysis, Systematic review, Distress, Quality of life, Functioning

## Abstract

**Background:**

The effect of cognitive behavioural therapy for psychosis (CBTp) on the core symptoms of schizophrenia has proven contentious, with current meta-analyses finding at most only small effects. However, it has been suggested that the effects of CBTp in areas other than psychotic symptoms are at least as important and potentially benefit from the intervention.

**Method:**

We meta-analysed RCTs investigating the effectiveness of CBTp for functioning, distress and quality of life in individuals diagnosed with schizophrenia and related disorders. Data from 36 randomised controlled trials (RCTs) met our inclusion criteria- 27 assessing functioning (1579 participants); 8 for distress (465 participants); and 10 for quality of life (592 participants).

**Results:**

The pooled effect size for functioning was small but significant for the end-of-trial (0.25: 95% CI: 0.14 to 0.33); however, this became non-significant at follow-up (0.10 [95%CI -0.07 to 0.26]). Although a small benefit of CBT was evident for reducing distress (0.37: 95%CI 0.05 to 0.69), this became nonsignificant when adjusted for possible publication bias (0.18: 95%CI -0.12 to 0.48). Finally, CBTp showed no benefit for improving quality of life (0.04: 95% CI: -0.12 to 0.19).

**Conclusions:**

CBTp has a small therapeutic effect on functioning at end-of-trial, although this benefit is not evident at follow-up. Although CBTp produced a small benefit on distress, this was subject to possible publication bias and became nonsignificant when adjusted. We found no evidence that CBTp increases quality of life post-intervention.

**Electronic supplementary material:**

The online version of this article (10.1186/s40359-018-0243-2) contains supplementary material, which is available to authorized users.

## Background

The first use of cognitive therapy to help people with schizophrenia was in 1952 [1]. Beginning somewhat later, with Kuipers et al. [2^*^], over 60 randomised controlled trials (RCTs) have subsequently examined the efficacy of Cognitive Behavioural Therapy for psychosis (CBTp). These trials have typically looked at the effectiveness of CBTp in improving the core symptoms of schizophrenia i.e., positive symptoms, or delusions and hallucinations measured separately, and in some cases negative symptoms. Recent meta-analyses of these trials have converged on finding symptomatic improvement that is in the *small* range (e.g. [3–9]. The most comprehensive of these meta-analyses - that of Jauhar et al. [7] - additionally found no effectiveness against positive symptoms in trials with blinded outcome assessments.

Over a decade ago, Birchwood and Trower [10] introduced the phrase ‘quasi-neuroleptic’ to describe the symptom-focused approach of CBTp. They argued that this view of CBTp was inappropriate and that the intervention was more likely to have a distinctive profile of effects that are complementary to rather than substituting for drug treatment. Such a view appears to be reflected in the two principal clinical guidelines in use in the UK, the National Institute for Care and Health Excellence (NICE) and the Scottish Intercollegiate Guidelines Network (SIGN). Thus, NICE [11] states that “The aims of psychological & psychosocial interventions in psychosis & schizophrenia are numerous. These should include interventions to improve symptoms but also those that address vulnerability, which are embedded in developmental processes. The aims, therefore, include: reduction of distress associated with psychosis symptoms… promoting social and educational recovery; reducing depression and social anxiety … and relapse prevention (p.32).” Similarly, SIGN [12] states: “The aim [of CBTp] is to help the individual normalise and make sense of their psychotic experiences, and to reduce the associated distress and impact on functioning (p.55)”. Similar sentiments are expressed in guidelines from elsewhere in the world, e.g. the Royal Australian and New Zealand College of Psychiatrists [13].

Nevertheless, the effect of CBTp on non-symptomatic outcomes in schizophrenia has been relatively less investigated than its effect on symptoms. Nearly 10 years ago, Wykes et al. [14] carried out a series of meta-analyses that included 15 trials which evaluated functioning. The pooled effect size was significant (Glass’s Δ = 0.38: 95% CI 0.15 to 0.60); however, analysing the trials by study quality (as measured using a unitary scale for this) revealed a large and significant difference in effect size between high and low-quality trials (0.15 vs. 0.51). They did not examine effect sizes for any follow-up period. Several meta-analyses of functioning were also carried out by the National Collaborating Centre for Mental Health (NCCMH) (http://www.rcpsych.ac.uk/workinpsychiatry/nccmh.aspx) for the purposes of the 2009 NICE guideline. These analyses assessed data relating to specific functioning scales and for all scales combined; examining effects at end-of-treatment and follow-up as well as against ‘treatment as usual’ (TAU) or other active controls (such as befriending or supportive counselling). The standardised mean difference (SMD) revealed that CBTp had no significant impact on functioning compared to TAU (K = 6: - 0.14, 95% CI -0.45 to 0.17), but at 12-month follow up was marginally significant (K = 4: -0.20, 95% CI-0.41 to − 0.00). When CBTp was contrasted with active controls, a medium effect emerged at the end-of-treatment (K = 3: SMD -0.50, 95% CI -0.84 to − 0.16); there was no meta-analysis against active controls at follow-up. The small numbers of trials analysed however, limits the reliability of findings from some of the NICE meta-analyses. The other main limiting factor concerning the meta-analyses by Wykes et al. [14] and NICE [11], is that the data in both are now a decade old.

NICE [11] also reported on a small number of trials measuring quality of life and found no significant advantage for CBTp compared to supportive counselling at the end of treatment (K = 3) (SMD 0.01, 95% CI –0.19 to 0.21) or for follow-up at either 52 weeks (K = 2; SMD -0.18, 95% CI -0.10 to 0.47) or 78 weeks (K = 1; SMD 0.40, 95% CI -0.17 to 0.98). In their Cochrane review of CBTp versus other psychosocial interventions, Jones et al. [6] included only one trial that examined quality of life [15] and no differential effect of CBTp was found either at end of treatment or follow-up in this trial. No meta-analysis appears to have examined the effects of CBTp on distress.

The aim of the series of meta-analyses reported here was to determine whether evidence shows that CBTp improves aspects of the patient experience beyond symptom-reduction. Based on there being enough trials to permit meaningful pooling of data, we selected three outcome variables: functioning, distress and quality of life.

## Method

We initially considered the 52 RCTs retreived by Jauhar et al. (2014), which covered the period of 1993 (the date of the first published trial of cognitive behavioural therapy in schizophrenia) to March 2013. We also searched the trials previously excluded by Jauhar et al. These studies were supplemented with a systematic search of the literature using PubMED and Scopus to identify RCTs of CBTp between the dates of March 2013 and April 2018. Searches were unrestricted regarding language and whether material was published or unpublished. We also searched through reference sections of papers that were considered eligible. Multiple searches were conducted using the following terms and combinations of terms:

“Cognitive Behavioural Therapy” AND “Psychosis” AND “Randomised controlled trial”.

“Cognitive Behavio*” AND “Psychosis” AND “Randomi*”.

“Cognitive Behavio*” AND “Psychosis” AND “RCT”.

“CBT” AND “Psychosis” AND “RCT”.

“CBT” AND “Psychosis” AND “Randomi*”.

“Cognitive Behavio*” AND “schizo*”.

“CBT” AND “Schizo*”.

“Cognitive Behavio*” AND “Schizo*” AND “RCT”.

“Cognitive Behavio*” AND “Schizo*” AND “Random*”.

“CBT” AND “Schizo*” AND “Randomi*”.

“CBT” AND “Schizo*” AND “RCT”.

This search produced a further 16 studies. All 69 studies were then hand-searched by one of us (ND) for the outcome measures of interest and counter-checked by another (KRL).

Our inclusion criteria paralleled those used by Jauhar et al. [7], Wykes et al. [14], NICE [11] and the Cochrane Collaboration [6]. Thus, studies were included if a majority of the patients had a diagnosis of schizophrenia, schizoaffective or non-affective functional psychosis, either made clinically or according to diagnostic criteria. Trials could use any measure of functioning, distress or quality of life (for details, see below). Studies also had to include a parallel control group of any type, i.e. waitlist, TAU or an intervention designed to control for the non-specific effects of psychotherapy. We excluded non-randomised trials and those which used inappropriate randomisation methods (e.g. allocation by alternation or by availability of the intervention). The four non-randomised trials that were located all also used non-blinded outcome assessment and were low in overall quality (see [16–19]).

Determination of what types of therapy constituted CBTp was relatively broad and followed Jauhar et al. [7] – those that incorporated additional elements of therapy, such as motivational interviewing, family engagement, behaviour therapy and social skills training, were also included. Following previous meta-analyses, we did not include studies that delivered CBT as part of a multicomponent package of care that involved several other interventions (sometimes referred to as integrated treatment or similar). We included trials using both individual and group CBTp.

### Data extraction

For functioning, trials used a variety of clinician-assessed rating scales which included: the Global Assessment of Functioning scale (GAF: [20]); the Social and Occupational Functioning Assessment Scale (SOFAS: [21]); the Global Assessment Scale (GAS: [22]); the Multnomah Community Ability Scale (MCAS: [23]); and the Life Skills Profile (LSP: [24]). Other scales considered to be includable were the Social Functioning Scale (SFS: [25]), the Role Functioning Scale (RFS: [26]), the Social Behaviour Schedule (SBS: [27]), the Independent Living Skills Survey (ILSS: [28]), and the Personal and Social Performance Scale (PSP: [29]).

Studies were included if they measured the distress associated with the symptoms of psychosis. Outcomes relating to depression and anxiety alone were not included as these were considered to represent symptomatic measures. Where articles provided more than one outcome measure for distress, ‘total distress’ scores were used. Measures included: the ‘distress’ domain within the Psychotic Symptom Rating Scale (PSYRATS: [30]); the Global Severity Index (GSI: [31]); and a questionnaire using a Likert scale ([32^*^]: On a scale from 0 to 10, how bothered are you when you experience (specific hallucination) [or think about (specific delusion)]?).

The quality of life measures used in trials included: the Quality of life scale (QLS: [33]); the World Health Organisation Quality Of Life Scale (WHOQOL-BREF: [34]); the Quality of life, Enjoyment and Satisfaction Questionnaire (Q-LES-Q: [35]); the Modular System for quality of life (MSQoL: [36]); and the Manchester Short Assessment of Quality of Life (MANSA: [37]).

#### Meta-analysis

Pooled effect sizes for the data were created using Comprehensive Meta-analysis, version 2 [38]. A random-effects model was used in all analyses. Effect sizes were derived from the post-intervention (or follow-up) scores using Hedges *g* (i.e. the standardized mean difference using group means divided by the pooled standard deviation: Eq. 1) and corrected for the tendency towards overestimation in small studies ([39] Eq. 2). When these data were not available in a paper, authors were contacted. Effect sizes are described using Cohen’s convention: an effect size of 0.20 was considered small, 0.50 moderate, and 0.80 large.

1 $$ smd=\frac{M_1-{M}_2}{SD\kern0.5em pooled} $$

2 $$ 1-\frac{3}{4N-1} $$

Heterogeneity was examined with *Q* and I^2^ statistics. An I^2^ value of 0–40% suggests that heterogeneity may not be important, 30–60% may represent moderate heterogeneity, 50–90% may represent substantial heterogeneity, and 75–100% may represent considerable heterogeneity (see [40]). Publication bias was examined using Duval and Tweedie’s [41] trim and fill technique, which aims to estimate the number of missing studies within an analysis and the effect that those studies might have on outcomes. Moderator analyses, where feasible, followed Jauhar et al. [7] and so, included comparisons of blind vs non-blind outcome-assessment and the use of active control vs treatment as usual. The latter categorical comparisons were conducted using a method analogous to ANOVA.

## Results

**Fig. 1:**
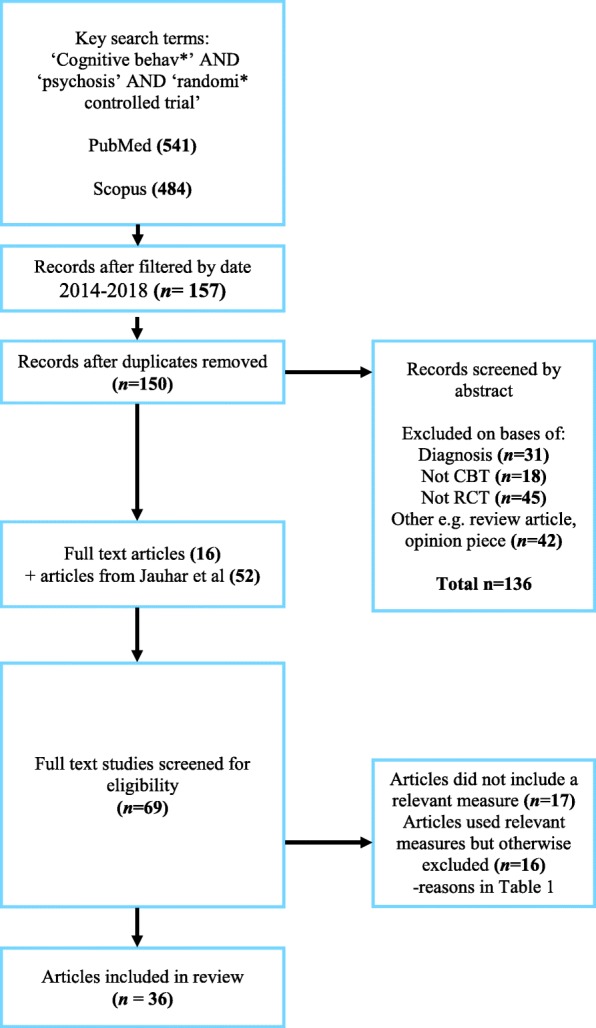
Flow chart outlining study selection

Thirty-six RCTs (37 samples) met our inclusion criteria (See Fig. 1), some measuring more than one outcome. Twenty-six samples assessed functioning, 8 assessed distress and 10 quality of life. See Table 1 for excluded studies and main reason for exclusion.

**Table 1:** Studies assessing outcomes but excluded with reasons

Study	Measure	Excluded on the basis that
Tarrier et al [42]	F	Did not obtainable/waitlist control was not a parallel group
Garety et al [16]	D	Non-randomised
Barrowclough et al [43]	F	Patients with comorbid substance abuse
Jenner et al [44]	F, QoL, D	CBT intervention was multimodal
Wiersma et al [45]	F, QoL	CBT intervention was multimodal
Grawe et al [46]	F	CBT intervention was multimodal
Jackson et al [17]	F, QoL	Non-randomised
Zimmer et al [47]	F, QoL	CBT intervention was multimodal
Gleeson et al [48]	F, QoL	CBT intervention was multimodal
Barrowclough et al [49]	F	Patients had comorbid substance abuse
Peters et al [50]	F	Patients described as ‘experiencing psychosis’, unable to confirm proportion with schizophrenia spectrum diagnoses
Mortan et al [18]	D	Non-randomised, small samples (CBT = 6 TAU = 5)
Grant et al. [51]	F	Data not obtainable
Drake et al [52]	F	All participants received CBT
Zanello et al [19]	F, QoL	Non-randomised, no control group
Waller et al [53]	D	Intervention not CBT

*Note.* D = distress, F = Functioning, QoL = Quality of life

### Functioning

Functioning was assessed in 25 trials (with 26 samples: see Additional file 1) providing a total of 1579 participants (780 received CBTp and 799 were in the control condition). Of the 26 samples, 17 compared CBTp to treatment as usual (TAU), while the remaining 9 compared it to another intervention (psychoeducation, befriending, cognitive remediation, social activity therapy, supportive therapy, goal focused supportive contact). The majority of studies used individual therapy (22/25 - only [54^*^–56^*^], and used group therapy).

**Fig. 2:**
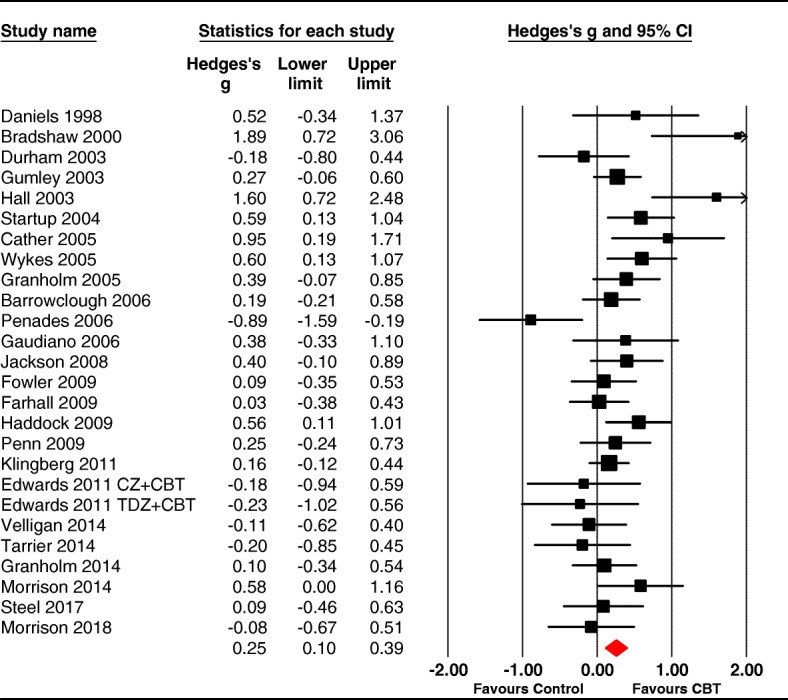
Forest plot for post-intervention scores on functioning. *Note.* Edwards et al. [58^*^] had intervention groups (Clozapine + CBT [CZ + CBT] and Thioridazine + CBT [TDZ + CBT] and two control groups i.e. Clozapine and Thioridazine respectively

The pooled effect size for functioning across 26 samples was 0.25 (95%CI: 0.14 to 0.33, *p <* .001, positive sign indicates CBTp better than control). The studies were moderately heterogeneous (*Q* [25] = 50.66, *p* < .001) with an I^2^ value of 50.66 (see forest plot in Fig. 2). Duval and Tweedie’s Trim and Fill [41] analysis revealed no evidence of publication bias. We re-ran the analysis removing one outlier trial [57^*^], which was the only one that revealed significantly worse functioning post CBT – this increased the effect size to 0.28 (95%CI .15 to .41) *p* < .001; Q [24] =39.52, *p* = .02, I^2^ = 39.27.

**Fig. 3:**
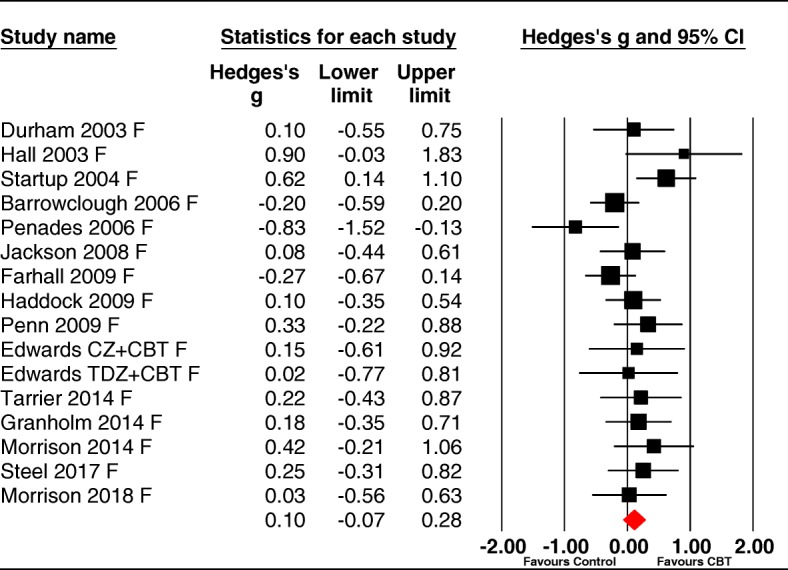
Forest plot for follow-up scores on functioning. *Note. F = follow-up*

#### Blind vs nonblind assessment

We compared 19 studies where assessors were blinded (masked) to treatment condition with 7 where assessment was not blinded (unmasked) to the treatment group. The unmasked trials revealed a small and significant effect size of 0.29 (95% CI: 0.10 to 0.48, *p* < .001); and the studies had low nonsignificant heterogeneity (Q = 6.94 [6], *p* = .33: I^2^ = 13.59). The masked trials revealed a small significant effect size of 0.22 (95% CI: 0.02 to 0.42, *p* = .03); these 19 studies were moderately heterogeneous (Q = 58.45 [18], *p* < .001; I^2^ = 58.45).

#### Active versus non-active control

We compared 19 trials using treatment as usual (TAU) as a control versus 7 trials using active control conditions. The effect size for TAU was significant at 0.26 (95%CI .08 to .43), *p* = .01; and showed low-moderate heterogeneity (Q = 34.83, df = 18, p = .01; I^2^ = 47.65). The effect size for trials with an active control was nonsignificant at 0.22 (95%CI -0.07 to 0.52, *p* = .14); and showed moderate heterogeneity (Q = 16.25, df = 6, *p* = .012; I^2^ = 63.07). The effect sizes from trials using TAU and active control did not significantly differ (Q = 0.03, df = 1, *p* = .86).

#### Follow-up

Follow-up data were available in 16 of the trials, with a median follow-up time of 12 months (range 3–18 months). Follow-up assessments involved 792 participants (393 CBTp and 399 controls) and retention was high with over 91% of the CBT and control participants examined at end-of-trial being assessed at follow-up.

The pooled effect size for CBTp on functioning at follow-up was nonsignificant 0.10 [95%CI -0.07 to 0.28], *p* = .23 (see Fig. 3). The samples showed low heterogeneity (Q = 21.78, df = 15, *p* = .11; I^2^ = 31.12). Most trials used blind assessment (K = 13: g = 0.12–0.08 to 0.32) and did not differ significantly in effect size (Q = 0.14, df = 1, *p* = .71) from nonblind trials (K = 3 g = 0.04–0.33 to 0.42) with both being nonsignificant.

### Distress

Distress was analysed in 8 studies (see Additional file 2) with a total sample size of 465 (235 receiving CBTp and 230 in control conditions). Of these studies, 7 were against a treatment as usual (TAU) and 1 was against a waitlist control. Most trials (7/8) used individual therapy with only [59^*^] using group therapy.

**Fig. 4:**
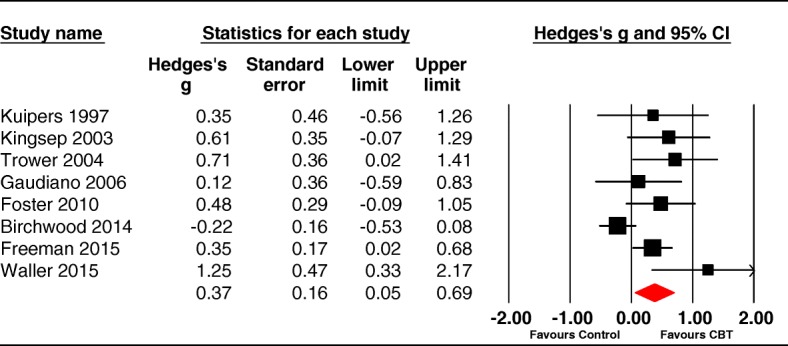
Forest plot for post-intervention scores on distress

The pooled effect size was significant at 0.37 (95% CI 0.05 to 0.69, *p* = .02). The studies were heterogeneous (Q (7) = 17.27, *p* = .01) with an I^2^ value of 60.51 suggesting moderate-high levels of true heterogeneity amongst the studies. The forest plot is shown in Fig. 4.

**Fig. 5:**
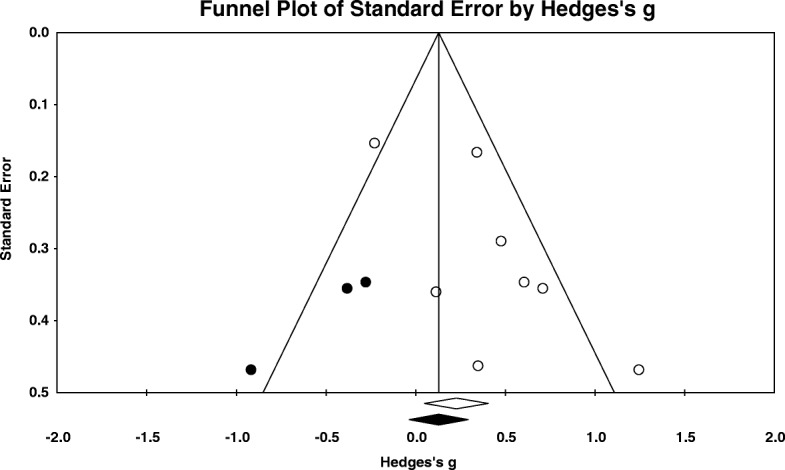
Funnel plot for distress (white dots are published trials & black dots imputed missing trials)

Duval and Tweedie’s trim and fill bias analysis [41] imputed 3 trials (see Fig. 5). When the meta-analysis was adjusted for this potential bias, the new effect size reduced and became nonsignificant (*g* = 0.18, 95% CI: -0.12 to 0.48).

Most trials were non-blind and these showed a significant distress reduction (K = 6, g = 0.43[95% CI 0.20 to 0.66]); however, the two blind trials [60^*^, 61^*^] produced a nonsignificant effect (0.19 [95% CI -0.72 to 1.10]).

### Quality of life

Quality of life was assessed in 10 samples from 9 trials (see Additional file 3) with a total sample size of 592 (293 received CBTp and 299 in the control condition. Of these studies, 1 was against an active control condition (psychoeducation/befriending), 7 were against a treatment as usual (TAU condition), and 2 were against a waitlist control. Three trials used group therapy ([59^*^, 62^*^, 63^*^], and) – the remaining 7 samples used individual therapy.

**Fig. 6:**
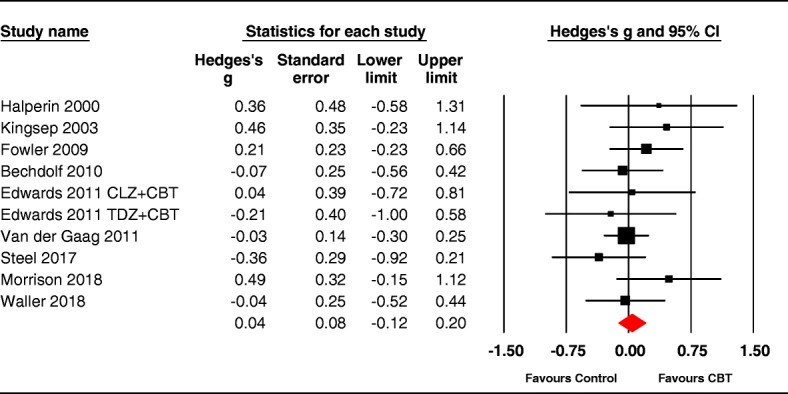
Forest plot for post-intervention scores on quality of life

CBTp had no significant impact on quality of life, with an effect size close to zero at 0.04 (95% CI: -0.12 to 0.19, *p* = .66). The studies were not heterogeneous (*Q* (9) = 7.19, *p* = .62) with an I^2^ value of 0. The forest plot in Fig. 6 presents the effect sizes for each trial, showing that none of the individual trials significantly improved QoL; both group (K = 3 g = 0.15 95% CI -0.22 to 0.51) and individual therapy were nonsignificant (K = 7, g = 0.01 95% CI -0.17 to 0.19) and I^2^ was zero in both.

When publication bias was examined, Duval and Tweedie’s trim and fill [41] imputed 1 missing effect size. With the analysis adjusted for this, the new effect size was reduced slightly (g = 0.01, 95% CI: -0.15 to 0.16).

The five trials examining QoL under blind conditions had a nonsignificant mean effect size of 0.06 [95% CI -0.24 to 0.36, *p* = .69], as did the three trials assessing QoL without blinding (0.16 [95%CI -0.20 to 0.52] *p* = .39); two further studies were unclear about blinding ([63, 64^*^] was presented blind, however raters correctly guessed 70% of the group assignments).

## Discussion

As noted in the introduction, while more than a dozen meta-analyses have examined whether CBTp reduces the positive and negative symptoms of schizophrenia, non-symptomatic outcomes have been somewhat neglected. Two previous meta-analyses – both now a decade old – have examined the impact of CBTp on functioning [11, 14] but ours is the first to examine the impact of CBTp across a range of non-symptomatic outcomes, including: functioning at end-of trial and follow-up and the impact on quality of life and distress. Although a small benefit of CBTp for functioning emerged at end-of-trial, this was non-significant at follow-up. In 8 trials, CBT was found to produce a small significant reduction in distress; however, evidence of potential publication bias led to the imputing of 3 studies, halving the effect size and making it non-significant. The effect was also moderated by blinding – significant distress reduction was only found in trials using non-blind outcome assessment. Quality of life was unaffected by CBTp and indeed, none of 10 samples documented a significant benefit.

With respect to functioning, our effect size of 0.25 (95% CI 0.14 to 0.33) for functioning is considerably smaller than the 0.38 effect size reported by Wykes et al. [14] in their meta-analysis of 15 trials - indeed, the Wykes et al. [14] effect size falls beyond the upper end of our 95% confidence intervals. One possible reason for this reducing effect size is that 12 of 14 RCTs published since Wykes et al’s 2008 [14] meta-analysis – and since NICE [11] published their current guidance on CBTp - have produced nonsignificant outcomes. Importantly, more recent studies also included large well-controlled trials (e.g [65^*^]). Furthermore, our analysis of follow-up data derived from 16 samples revealed that CBT did not significantly improve functioning. This latter finding contrasts with the findings reported by NICE; it seems likely that this reflects the fact that the current meta-analysis is much larger - involving four times as many trials. Our findings provide an important update on the multiple meta-analyses carried out for NICE (2009), which was on small numbers of trials and produced mixed findings. NICE have still failed to update their meta-analyses, which contain no trials post-2008; and so, it might seem an appropriate time to update their analyses and potentially, their recommendations given the findings here. The repeated decisions by NICE to *not* update CG178 with any trials post-2008 has also been remarked upon in meta-analyses and indeed, by the Chair of SIGN [7, 66].

With an effect size that was close to zero, we found no suggestion that CBTp improves quality of life in people diagnosed with schizophrenia. Our findings accord with earlier smaller analyses of quality of life by NICE [11] and the Cochrane Collaboration [6], both of which found no evidence of CBTp being efficacious for this outcome. Although the current number of trials remains quite small (K = 9 and 10 samples), we found little to suggest that missing trials or methodological factors - such as blinding or type of control group - were playing any role in this null finding. Indeed, every published trial has reported a nonsignificant effect of CBTp on quality of life; particularly noteworthy is one trial by van der Gaag et al. [64^*^] which had large numbers (109 CBTp and 97 controls) and an effect size of zero.

Despite CBTp being promoted as effective against distress by both NICE [11] and SIGN [12], this outcome has received surprisingly little interest from triallists. Only 8 in 67 RCTs that met our eligibility criteria reported distress as an outcome and this was always as a secondary measure. Although significant at 0.37, the effect size for distress was prone to potential publication bias and when adjusted for three potentially missing trials, became small and nonsignificant at 0.18. Also noteworthy is that several RCTs assessing distress had small samples and so their power to detect true (small) effects is likely to be low. Following Button et al. [67], it is possible to derive the median statistical power of each study in the meta-analyses to obtain the overall effect size (using the mean effect sizes as the best estimate of likely true effect size). Doing this revealed that the power in CBTp trials assessing distress was low at .22, whereas those for quality of life and functioning were somewhat better but still underpowered at .50 and .64 respectively. The low level of power also accords with the evidence of potential publication bias in trials measuring distress; and may reflect the publishing of unreliable small trials with positive, but not negative results. Future studies of distress would need four times the current mean sample size of 40 per group to reliably detect the effect size reported in existing trials. Only one trial, that of Birchwood et al. [61^*^], comes close to the sample size required, and this found *increased* distress following CBTp. Clearly adequate powering is essential in future trials – not only to accurately ascertain if CBTp reduces distress, but to eliminate any possibly that it may increase distress in some patients.

## Conclusions

Our meta-analysis is the first to assess whether CBTp improves quality of life or reduces distress in individuals diagnosed with schizophrenia. We also present an updated meta-analysis assessing the impact of CBTp on functioning. On current evidence CBTp leads to a small improvement in functioning which, however, is not sustained. The case for beneficial effects on quality of life and distress appear, from studies to date, to be weak. Overall, the three meta-analyses performed provide only equivocal support for the non-quasi-neuroleptic hypothesis of CBTp, with its emphasis on these outcomes.

## Additional files

Additional file 1: Randomised Controlled Trials that measured functioning as an outcome (DOCX 20 kb)

Additional file 2: Randomised controlled trials of CBTp that measured distress as an outcome measure (DOCX 17 kb)

Additional file 3: Randomised Controlled Trials that measured quality of life as an outcome measure (DOCX 23 kb)

## Abbreviations

95%CI: 95% Confidence Intervals
CBTp: Cognitive Behavioural Therapy for psychosis
GAF: Global Assessment of Functioning scale
GAS: Global Assessment Scale
GSI: Global Severity Index
ILSS: Independent Living Skills Survey
LSP: Life Skills Profile
MANSA: Manchester Short Assessment of Quality of Life
MCAS: Multnomah Community Ability Scale
MSQoL: Modular System for quality of life
NCCMH: National Collaborating Centre for Mental Health
NICE: National Institute for Care and Health Excellence
PSP: Personal and Social Performance Scale
PSYRATS: Psychotic Symptom Rating Scale
Q-LES-Q: Quality of life, Enjoyment and Satisfaction Questionnaire
QLS: Quality of life scale
RCT: Randomised Controlled Trial
RFS: Role Functioning Scale
SBS: Social Behaviour Schedule
SFS: Social Functioning Scale
SIGN: Scottish Intercollegiate Guidelines Network
SMD: Standardised mean difference
SOFAS: Social and Occupational Functioning Assessment Scale
TAU: Treatment as usual
WHOQOL-BREF: World Health Organisation Quality Of Life Scale
