# Supplementary material for: Cognitive Behavioural Therapy for schizophrenia - outcomes for functioning, distress and quality of life: a meta-analysis
Source: BMC Psychol. 2018 Jul 17;6:32. doi: 10.1186/s40359-018-0243-2 (PMC6050679; doi:10.1186/s40359-018-0243-2)
Supplement: Supplementary file 1 — Randomised Controlled Trials that measured functioning as an outcome (DOCX 20 kb) [file 40359_2018_243_MOESM1_ESM.docx]

**Additional File 1**

Randomised Controlled Trials that measured functioning as an outcome

| Study | Sample | Intervention | | Control | | Instrument | Masked |
| --- | --- | --- | --- | --- | --- | --- | --- |
|  |  |  |  |  |  |  |  |
| Daniels [54] | Schizophrenia | Interactive Behavioral Training (group) | 10 | Waitlist | 10 | GAF | Y |
| Bradshaw [68] | Schizophrenia | CBT | 12 | TAU | 12 | RFS | Y |
| Durham [69] | Schizophrenia or delusional disorder | CBT | 22 | TAU | 21 | GAS | Y |
| Gumley [70] | Schizophrenia | CBT | 72 | TAU | 72 | SFS | N |
| Hall [71] | Individuals experiencing psychosis who scored poorly on a self-esteem measure | CBT | 12 | TAU | 13 | SFS | Y |
| Startup [72] | Schizophrenia | CBT | 47 | TAU | 43 | GAF | Y |
| Cather [73] | Schizophrenia | Functional CBT | 15 | Psychoeducation | 13 | SFS | N |
| Wykes [55] | Schizophrenia | Group CBT for voices | 45 | TAU | 40 | SBS | N |
| Granholm [74] | Schizophrenia  (older outpatients) | CBT social skills training | 37 | TAU | 39 | ILSS | Y |
| Barrowclough [56] | Schizophrenia | Group CBT | 57 | TAU | 56 | GAF | Y |
| Penadés [57] | Schizophrenia | CBT | 20 | Cognitive Remediation Therapy | 20 | LSP | Y |
| Guadiano & Herbert [32] | Schizophrenia | ACT | 19 | TAU | 21 | SDS | N |
| Jackson [75] | First episode psychosis | Acute Cognitive Therapy for Early Psychosis (ACE) | 31 | Befriending | 31 | SOFAS | Y |
| Fowler [76] | Early psychosis and social disability | CBT | 35 | TAU | 42 | SOFAS, | Y |
| Farhall [77] | Schizophrenia | CBT | 45 | TAU | 49 | LSP | N |
| Haddock [78] | Schizophrenia | CBT | 38 | SAT | 39 | GAF | Y |
| Penn [79] | Schizophrenia | CBT for auditory hallucinations | 33 | Supportive Therapy | 32 | SFS | N |
| Klingberg [65] | Schizophrenia | CBT | 99 | Cognitive Remediation | 99 | GAF | Y |
| Edwards [58] | First episode psychosis | CBT + Clozapine | 11 | Clozapine | 14 | GAF | Y |
| Edwards [58] | First episode psychosis | CBT + thioridazine, | 12 | thioridazine | 11 | GAF | Y |
| Velligan [80] | Schizophrenia | CBT | 37 | TAU | 37 | MCAS | Y |
| Tarrier [81] | Schizophrenia | Cognitive Behavioural Prevention of Suicide in psychosis protocol | 17 | TAU | 18 | GAF | Y |
| Granholm [88] | Schizophrenia | Cognitive Behavioural Social Skills Training | 35 | Goal-focused supportive contact | 44 | ILSS | N |
| Morrison [82] | Schizophrenia (not taking antipsychotics) | CBT | 37 | TAU | 37 | PSP | Y |
| Steel [83] | schizophrenia, schizo-affective disorder or schizophreniform disorder, and met DSM-IV criteria for PTSD | CBT | 25 | TAU | 25 | GAF | Y |
| Morrison [84] | schizophrenia, schizo-affective disorder or delusional disorder who met criteria for early intervention | CBT+Clozapine | 21 | Clozapine | 22 | PSP | Y |
|  |  |  |  |  |  |  |  |

**Note.** Global Assessment of Functioning scale (GAF); Social and Occupational Functioning Assessment Scale (SOFAS: Goldman et al. 1992); Global Assessment Scale (GAS); Multnomah Community Ability Scale (MCAS); the Life Skills Profile (LSP);Social Functioning Scale (SFS); Role Functioning Scale (RFS); Social Behaviour Schedule (SBS); Independent Living Skills Survey (ILSS); and the Personal and Social Performance Scale (PSP); Sheenan Disability Scale (SDS).
