# Supplementary material for: Cognitive Behavioural Therapy for schizophrenia - outcomes for functioning, distress and quality of life: a meta-analysis
Source: BMC Psychol. 2018 Jul 17;6:32. doi: 10.1186/s40359-018-0243-2 (PMC6050679; doi:10.1186/s40359-018-0243-2)
Supplement: Supplementary file 2 — Randomised controlled trials of CBTp that measured distress as an outcome measure (DOCX 17 kb) [file 40359_2018_243_MOESM2_ESM.docx]

**Additional File 2**

Randomised controlled trials of CBTp that measured distress as an outcome measure

| Study | Diagnosis/Sample | Intervention | | Control | | Instrument |
| --- | --- | --- | --- | --- | --- | --- |
|  |  |  |  |  |  |  |
| Kuipers [2] | Schizophrenia | Individualised CBT for psychosis | 28 |  | 32 | Self-report delusional distress |
| Kingsep [59] | Comorbid Schizophrenia and Anxiety | Cognitive Behavioural Group Therapy (CBGT) | 16 | Waitlist control | 17 | GSI |
| Tower [60] | Schizophrenia | Cognitive therapy for command hallucinations | 18 | TAU | 20 | PSYRATS (distress score) |
| Gaudiano [32] | Individuals experiencing psychosis | Mindfulness/Acceptance-based CBT | 19 | Enhanced TAU | 21 | Self-report measure |
| Foster [85] | Schizophrenia, schizoaffective disorder or delusional disorder | Cognitive Behavioural Worry Intervention | 12 | TAU | 12 | PSYRATS (distress score) |
| Birchwood [61] | Voice hearers | Cognitive therapy for command hallucinations | 98 | TAU | 99 | PSYRATS (distress score) |
| Freeman [86] | Persistent persecutory delusions | CBT for worry | 73 | TAU | 77 | PSYRATS (distress score) |
| Waller [87] | Participants with distressing persecutory delusions and schizophrenia | Thinking-well intervention | 20 | TAU | 11 | PSYRATS (distress score) |
|  |  |  |  |  |  |  |

**Note** Psychotic Symptom Rating Scale (PSYRATS); Global Severity Index (GSI)
