# Supplementary material for: Cognitive Behavioural Therapy for schizophrenia - outcomes for functioning, distress and quality of life: a meta-analysis
Source: BMC Psychol. 2018 Jul 17;6:32. doi: 10.1186/s40359-018-0243-2 (PMC6050679; doi:10.1186/s40359-018-0243-2)
Supplement: Supplementary file 3 — Randomised Controlled Trials that measured quality of life as an outcome measure (DOCX 23 kb) [file 40359_2018_243_MOESM3_ESM.docx]

**Additional File 3**

Randomised Controlled Trials that measured quality of life as an outcome measure

| Study | Diagnosis/Sample | Intervention | |  | Control | | Instrument |
| --- | --- | --- | --- | --- | --- | --- | --- |
|  |  |  |  |  |  |  |  |
| Halperin [62] | Comorbid Schizophrenia and Anxiety | Group based CBT | 7 |  | Waitlist control | 9 | Q-LES-Q |
| Kingsep [59] | Comorbid Schizophrenia and Anxiety | Cognitive Behavioural Group Therapy (CBGT) | 16 |  | Waitlist control | 17 | Q-LES-Q |
| Fowler [76] | Early psychosis and social disability | CBT + TAU | 35 |  | TAU | 42 | QLS |
| Bechdolf [63] | Schizophrenia | Group CBT | 40 |  | Psychoeducation | 48 | MSQoL |
| Edwards [58] | First episode psychosis | CBT + Clozapine | 11 |  | Clozapine | 14 | QLS |
| Edwards [58] | First episode psychosis | CBT + thioridazine, | 12 |  | thioridazine, | 11 | QLS |
| Van der Gaag [64] | Schizophrenia | CBT | 109 |  | TAU | 97 | WHO |
| Steel [83] | schizophrenia, schizo-affective disorder or schizophreniform disorder, and met DSM-IV criteria for PTSD | CBT | 25 |  | TAU | 25 | QLS |
| Waller [53] | Schizophrenia spectrum disorder | CBT targeting personalised recovery goal | 30 |  | TAU | 35 | MANSA |
| Morrison [84] | schizophrenia, schizo-affective disorder or delusional disorder who met criteria for early intervention | CBT+Clozapine | 21 |  | TAU (Clozapine) | 22 | WHO |

**Note**. Quality of life scale (QLS); World Health Organisation Quality Of Life Scale (WHO); Quality of Life, Enjoyment and Satisfaction Questionnaire (Q-LES-Q); Modular System for Quality of Life (MSQoL); Manchester Short Assessment of Quality of Life (MANSA)
